# Antiproliferative Activity, Antioxidant Capacity and Tannin Content in Plants of Semi-Arid Northeastern Brazil

**DOI:** 10.3390/molecules15128534

**Published:** 2010-11-24

**Authors:** Joabe Gomes de Melo, Thiago Antônio de Sousa Araújo, Valérium Thijan Nobre de Almeida e Castro, Daniela Lyra de Vasconcelos Cabral, Maria do Desterro Rodrigues, Silene Carneiro do Nascimento, Elba Lúcia Cavalcanti de Amorim, Ulysses Paulino de Albuquerque

**Affiliations:** 1 Biology Department, Pernambuco Federal University, Rua Dom Manoel de Medeiros, s/n, Recife, PE, Brazil; E-Mail: jgm2005@ig.com.br; 2 Department of Pharmaceutical Sciences, Health Sciences Center, Pernambuco Federal University, Av., Prof. Arthur de Sá, s/n, 50740-521, Recife, PE, Brazil; E-Mails: thiagocaruaru@hotmail.com (T.A.S.A.); valerium_castro@hotmail.com (V.T.N.A.C.); cabral803@hotmail.com (D.L.V.C.); elba@ufpe.br (E.L.C.A.); 3 Department of Antibiotics, Pernambuco Federal University, Av. Prof. Arthur de Sá, s/n, 50740-521, Recife, PE, Brazil; E-Mails: mdrodrigues@yahoo.com.br (M.D.R.); silenen@yahoo.com.br (S.C.N.)

**Keywords:** Caatinga, antiproliferative, antioxidant, tannin

## Abstract

The objective of this study was to evaluate antiproliferative activity, antioxidant capacity and tannin content in plants from semi-arid northeastern Brazil (Caatinga). For this study, we selected 14 species and we assayed the methanol extracts for antiproliferative activity against the HEp-2 (laryngeal cancer) and NCI-H292 (lung cancer) cell lines using the (3-[4,5-dimethylthiazol-2-yl]-2,5-diphenyltetrazole) (MTT) method. In addition, the antioxidant activity was evaluated with the DPPH (2,2-diphenyl-2-picrylhydrazyl) assay, and the tannin content was determined by the radial diffusion method. Plants with better antioxidant activity (expressed in a dose able to decrease the initial DPPH concentration by 50%, or IC_50_) and with higher levels of tannins were: *Poincianella pyramidalis* (42.95 ± 1.77 µg/mL IC_50_ and 8.17 ± 0.64 tannin content), *Jatropha mollissima* (54.09 ± 4.36µg/mL IC_50_ and 2.35 ± 0.08 tannin content) and *Anadenanthera colubrina* (73.24 ± 1.47 µg/mL IC_50_ and 4.41 ± 0.47 tannin content). Plants with enhanced antiproliferative activity (% living cells) were *Annona muricata* (24.94 ± 0.74 in NCI-H292), *Lantana camara* (25.8 ± 0.19 in NCI-H292), *Handroanthus impetiginosus* (41.8 ± 0.47 in NCI-H292) and *Mentzelia aspera* (45.61 ± 1.94 in HEp-2). For species with better antioxidant and antiproliferative activities, we suggest future *in vitro* and *in vivo* comparative studies with other pharmacological models, and to start a process of purification and identification of the possible molecule(s) responsible for the observed pharmacological activity. We believe that the flora of Brazilian semi-arid areas can be a valuable source of plants rich in tannins, cytotoxic compounds and antioxidant agents.

## 1. Introduction

Molecules derived from plants (e.g., vincristine, taxol and etoposide) have played an important role in cancer therapy and continue to be a promising source of new therapeutic agents [1]. For the discovery of new anticancer agents, herbal extracts are taken once the plant species are selected (usually based on random, chemosystematic, ecological and/or ethnobotanical criteria) and these are subsequently evaluated using several cancer cell lines. Next, extracts with high *in vitro* cytotoxic activity against tumor cells are prioritized for more in-depth studies such as evaluation of fractions, isolation of possible molecules that may be responsible for the cytotoxic activity, and even *in vivo* studies. 

Antioxidants are a group of substances that are useful for fighting cancer and other processes that potentially lead to diseases such as atherosclerosis, Alzheimer’s, Parkinson’s, diabetes, and heart disease [2]. Unlike cytotoxic agents that damage tumor cells, antioxidants act by preventing the onset of cancer during carcinogenesis, and they are generally beneficial to cells. Oxidants, such as reactive oxygen and nitrogen species that include the superoxide radical (O_2_^●^−), hydroxyl radical (OH^●^), hydroperoxyl radical (ROO^●^), peroxynitrite (^●^ONOO^−^), and nitric oxide (NO^●^), damage macromolecules, such as proteins, lipids, enzymes, and DNA [3]. To combat these radicals, living organisms produce enzymes (e.g., catalase, superoxide dismutase, and peroxidase) or rely on non-enzymatic molecules, such as cysteine, ascorbic acid, flavonoids, and vitamin K for protection [3].

Among the non-enzymatic compounds obtained from natural sources, phenols have received special attention due to their proven antioxidant capabilities [3]. Phenols derive from secondary metabolism and have a wide distribution in the plant kingdom with various functions in plants, such as chemical defense against herbivores and allelopathy [4,5]. Although phenolic compounds have been related to antioxidant activity, some studies have emphasized specific classes, such as the flavonoids and tannins [6,7].

The objective of this study was evaluate the antiproliferative activity, antioxidant capacity and tannin content in 14 plants from semi-arid northeastern Brazil. The plants selected for this study were collected in a semi-arid ecosystem region in northeastern Brazil called the Caatinga (dry forest). The Caatinga possesses vegetation consisting primarily of small woody plants [8]. Few pharmacological and phytochemical studies have been done on species of this ecosystem. However, the reported studies have yielded species with antioxidant activity [9,10] and high levels of tannins [11,12].

## 2. Results and Discussion

### 2.1. Antioxidant activity

Of the 14 plants tested (see Table 1), four stood out because they showed the lowest sample concentration required to reduce free radicals by 50% (IC_50_). These species were *Poincianella pyramidalis*, *Jatropha mollissima*, *Anadenanthera colubrina* and *Croton blanchetianus*. The positive control used in this assay was ascorbic acid, which showed an IC_50_ of 21.74 ± 3.23 µg. Overall, the IC_50_ values of the analyzed plants ranged from 42.95 to 1123.28 µg/mL. In a study performed by David *et al*. [10], where the DPPH radical capture activity of methanol extracts of 32 Caatinga plants, was also evaluated, the IC_50_s ranged from 0.3 to 25.1 mg/mL. 

According to the results, we can classify these 14 plants into three groups, based on the performance of the crude extract antioxidant activity: I - Good activity (IC_50_ <65 µg, value on average up to three times higher than the positive control); II-average activity (65 µg <IC_50_ <152 µg, value on average between 3-7 times higher than the positive control), III-low activity (IC_50_> 152 µg, value on average seven times higher than the positive control). Using this classification, two plants showed good activity, three plants had average activity and nine plants showed low activity (Table 1). 

### 2.2. Tannin content

Six plants were shown to contain tannins by the radial diffusion method. These were *Poincianella pyramidalis, Anadenanthera colubrina*, and *Amburana cearensis* among the trees species, *Jatropha mollissima* and *Croton blanchetianus* among the shrubs, and *Cyperus distans* among the herbs. Of the six plants that showed tannins by this test method, four presented the best antioxidant activity results. This suggests that tannins may be contributing to a better performance in the antioxidant activity tests. Although tannins in general exhibit antioxidant activity [7] specific research is needed to test the relationship between tannin content and antioxidant activity of Caatinga plants. 

### 2.3. Antiproliferative activities

The four plants that showed the most cytotoxic effects (lower percentage of living cells) were *Annona muricata* (54.92 ± 1.44 in HEp-2 and 24.94 ± 0.74 in NCI-H292), *Lantana camara* (55.98 ± 0.74 in HEp-2 and 25.08 ± 0.19 in NCI-H292), *Handroanthus impetiginosus* (45.73 ± 2.19 in HEp-2 and 41.8 ± 0.47 in NCI-H292) and *Mentzelia aspera* (45.61 ± 1.94 in HEp-2 and 86.04 ± 0.35 in NCI-H292). Among the plants with higher proliferative activity (% living cells), *Jatropha mollissima* (142.06 ± 5.06 in HEp-2), *Cyperus distans* (132.09 ± 7.99 in HEp-2 and 102.03 ± 1.71 in NCI-H292), and *Anadenanthera colubrina* (117.32 ± 1.76 in HEp-2) stood out. The positive control used was a pharmaceutical formulation (injectable solution) based on etoposide that showed (% living cells) 42.9 ± 1.14 in the HEp-2 cell line and 47.88 ± 0.92 in the NCI-H292 in the concentration of 5 µg/mL of the product. 

According to the criteria used by the South-American Office for Anti-Cancer Drug Development (SOAD), samples that inhibit at least 50% growth (with a dose of 50 µg/mL in a cell line) are candidates for future studies face a panel of different strains of human cancer, and later, according to the result, a purification process guided by bioassays [13]. Since *Annona muricata*, *Lantana camara*, *Handroanthus impetiginosus* have studies evaluating the antitumor activity *in vitro* and/or *in vivo* [14,15,16,17,18,19], we are currently conducting specific studies on *Mentzelia aspera*.

## 3. Experimental

### 3.1. Plant selection

The 14 species employed in this study were selected from a survey of flora and herbaceous trees and shrubs found in a rural area in the municipality of Altinho (08°29′23″S and 36°03′34″W), in the state of Pernambuco (NE Brazil). From this sample, we performed a sampling of inventoried species, evaluated the cytotoxic activity *in vitro* and selected those with the best anti-proliferative and proliferative results.

The survey of the herbaceous flora was performed in three different anthropogenic zones: areas where *Zea mays* L. and *Opuntia ficus-indica* (L.) Mill. were under cultivation and a native pasture area. For each area, 100 1 × 1 m plots were sampled for a total of 300 plots. The survey was conducted between November 2007 and July 2008 and a total of 119 species were registered in these three areas. More details of the survey can be found in Santos *et al*. [20].

The floristic and phytosociological survey of the woody-shrubby layer was performed on a fragment of native vegetation located in a hilly area using the point-Quadrant method. For this method, three parallel lines of 500 meters, were laid out 10 meters apart. For each point, four vertices were created at an angle of 90 degrees, and individuals with a diameter ≥3 cm above ground level and closer to each vertex were sampled. A total of 150 points were demarcated and 600 individuals sampled for a total of 48 identified taxa. *Annona muricata,* an exotic species, was also included in this study due to its popularity in cancer treatment by local inhabitants in this region.

All material collected was identified by experts and incorporated into the Professor Vasconcelos Sobrinho Herbarium of the Universidade Federal Rural de Pernambuco and duplicates sent to the Herbarium Dardano de Andrade Lima (Agronomic Institute of Pernambuco) and to Sergio Tavares (Universidade Federal Rural de Pernambuco).

### 3.2. Preparation of extracts

To obtain the extracts used in the antioxidant and proliferative/antiproliferative activity, plant parts were placed at room temperature for dehydration, subsequently triturated and then the plants (10 g) were macerated with methanol (300 mL) for 72 hours. Extracts were filtered and the solvents removed under reduced pressure. For tannin content tests, triturated plant (100 mg) was macerated with water/methanol (1:1, 1 mL) for two hours [21].

### 3.3. Determination of tannins

The determination of tannins was performed by the radial diffusion method [21]. This method consists of the reaction between tannins and proteins in an agarose gel forming a measurable and visible ring. For this reaction, a solution of 50 mM acetic acid and 60 µM ascorbic acid were prepared and adjusted to pH 5.0 [21]. This solution was then used to prepare the gel by adding 1% agarose (type I, Sigma-Aldrich). This solution was then heated until complete agarose homogenization and the solution cooled to 45 °C. Next, 0.1% bovine serum albumin (BSA) fraction V free fatty acid (Sigma-Aldrich) was added. Quickly, the gel was distributed in aliquots of 10 mL in 9.0 cm in diameter Petri dishes. Four-millimeter diameter wells were made on the gel 2.0 cm apart and from the plate edges, each having a volumetric capacity of 8 µL. With the help of a micropipette, three successive aliquots of 8 µL of each extract were added to the wells. All samples were processed in authentic triplicates. 

To obtain the standard curve, an aqueous solution of tannic acid at a concentration of 25 mg/mL was prepared and aliquots of 2, 4, 8, 12, 16, 20, and 24 µL were placed in wells, in triplicate, and divided between more than one well whenever the aliquots were larger than the capacity of the well.

### 3.4. Antiproliferative activity

*In vitro* evaluation of antiproliferative activity was carried out on two cancer cell lines (HEp-2 and NCI-H292). NCI-H292 is a mucoepidermoid cell line derived from human lung carcinoma, and HEp-2 cells are derived from primary tumors of the human larynx. The HEp-2 and NCI-H292 cell lines were maintained in a suitable medium (Dulbecco’s modified Eagle’s Minimum Essential Medium [Sigma]) with the addition of 10% fetal bovine serum (Sigma) and 1% L-glutamine (200 mM). Cell viability was determined by 0.4% Trypan blue (Merck). Cell counting was performed on a Leitz inverted microscope using a hemocytometer. The cell suspensions were distributed in 96-well culture plates (198 μL in each well). These were incubated at 37 °C and 5% humidity in an appropriate incubator. After 24 h of incubation, the extracts were added (at a concentration of 50 µg/mL) and the plates again incubated at 37 °C [22].

After 72 hours, 3-[4,5-dimethylthiazol-2-yl]-2,5-difeniltetrazole (MTT) bromide (25 μL) was added to each well at a concentration of 5 mg/mL in PBS. The plates were then left for two hours in an incubator (37 °C). Subsequently, the culture medium and MTT were removed by aspiration, and dimethylsulfoxide (100 μL) was added to each well to dissolve the crystals that formed [23]. To verify the percentage of inhibition, optical readings were performed on a Multiscan-type automatic plate reader at 595 nm. All measurements were performed in triplicate.

### 3.5. Quantification of antioxidant activity using the DPPH method (2,2-diphenyl-2-picrylhydrazyl)

Six different concentrations were prepared from the extracts (250, 200, 150, 100, 50, and 25 or 100, 50, 25, 20, 15, and 10 µg/mL) with the objective of obtaining an exponential curve. This variation depended on the antioxidant power of the extract, given that higher concentrations of certain species saturated the DPPH solution, leading to similar absorbance values and poor curve shape.

The protocol was adapted from Cotelle *et al*. [24] and McCune and Johns [25,26] and quantified the antioxidant activity using the (2,2-diphenyl-2-picrylhydrazyl) (DPPH) assay A 40-µM DPPH solution in methanol was prepared for this assay. Next, for each concentration, plant extract (0.5 mL) was removed and mixed with the DPPH standard solution (3.0 mL) in a test tube. After 30 minutes, the absorbance of this solution was read at 517 nm. A duplicate reading was performed for each concentration.

The positive control in this assay was ascorbic acid, used at concentrations of 5, 10, 15, 20, 25, 30, 40, and 50 µg/mL, and subjected to the same aforementioned procedures for the quantification of antioxidant activity.

With these different concentrations, the inhibitory concentration (IC50) was calculated, which corresponds to the concentration required to increase or decrease the initial DPPH concentration by 50%. To calculate the IC50, the concentrations of the samples and the positive controls (µg/mL) were plotted on the abscissa, and the percentage of the remaining DPPH (% DPPHREM) on the ordinate, to obtain a first-order exponential curve and an equation from which the effective concentration could be calculated.

## 4. Conclusions

In this work, we present data on the evaluation of antiproliferative activity, tannin content and antioxidant capacity in 14 plants from the northeastern semi-arid region of Brazil. For species with better antioxidant and antiproliferative activities, we suggest future comparative studies with other pharmacological models, *in vitro* and *in vivo*, and to start a process of purification and identification of possible molecule(s) responsible for the observed pharmacological activity. 

## Figures and Tables

**Table 1:** Tannin content, antioxidant activity, and antiproliferative activity of plants from a semi-arid region of Brazil.

Family/ *Species* (Voucher number)	Type/ part used	Antioxidant activity (IC50 in µg/mL)	Tannin content (mg/100 mg)	Percentage of living cells in the HEp-2 cell line (%)	Percentage of living cells in the NCI-H292 cell line (%)
**Annonaceae**					
*Annona muricata* L. (50480)	tree/ leaves	221.52 ± 16.12	ND	54.92 ± 1.44	24.94 ± 0.74
**Asteraceae**					
*Ageratum conyzoides* L. (50478)	herb/ aerial parts	340.17 ± 31.94	ND	81.45 ± 0.85	105.14 ± 3.34
*Delilia biflora* (L.) Kuntze (50477)	herb/ aerial parts	533.02 ± 31.92	ND	58.19 ± 2.49	77.37 ± 1.05
**Bignoniaceae**					
*Handroanthus impetiginosus* (Mart. ex DC.) Mattos (50481)	tree/ leaves	173.17 ± 16.56	ND	45.73 ± 2.19	41.8 ± 0.47
**Cyperaceae**					
*Cyperus distans* L. f. (50487)	herb/ aerial parts	258.42 ± 15.29	1.22 ± 0.02	132.09 ± 7.99	102.03 ± 1.71
**Euphorbiaceae**					
*Croton blanchetianus* Baill. (48667)	shrub/ leaves	94.41 ± 2.67	2.13 ± 0.09	103.56 ± 3.88	94.29 ± 3.96
*Jatropha mollissima* (Pohl) Baill. (48661)	shrub/ leaves	54.09 ± 4.36	2.35 ± 0.08	142.06 ± 5.06	88.32 ± 0.3
**Fabaceae**					
*Amburana cearensis* (Allemão) A.C. Sm. (50486)	tree/ leaves	203.14 ± 6.83	1.55 ± 0.11	94.58 ± 4.31	103.74 ± 1.32
*Anadenanthera colubrina* (Vell.) Brenan (48663)	tree/ leaves	73.24 ± 1.47	4.41 ± 0.47	117.32 ± 1.76	97.24 ± 0.89
*Poincianella pyramidalis* (Tul.) L. P. Queiroz (48662)	tree/ leaves	42.95 ± 1.77	8.17 ± 0.64	90.97 ± 0.94	105.46 ± 0.97
*Crotalaria incana* L. (50485)	shrub/ leaves	1123.28 ± 153.21	ND	115.39 ± 2.06	102.73 ± 2.23
*Senna occidentalis* (L.) Link (50484)	shrub/ leaves	628.27 ± 85.14	ND	103.37 ± 1.61	99.42 ± 0.77
Loasaceae					
*Mentzelia aspera* L. (50483)	herb/ aerial parts	911.5 ± 166.39	ND	45.61 ± 1.94	86.04 ± 0.35
Verbenaceae					
*Lantana camara* L. (50479)	shrub/ leaves	114.63 ± 6.16	ND	55.98 ± 0.74	25.08 ± 0.19

ND: not detected.
